# Mitochondrial respiratory dysfunctions of alveolar macrophages in interstitial lung disease: an exploratory study of bioenergetic and clinical links

**DOI:** 10.3389/fmed.2025.1719710

**Published:** 2026-01-22

**Authors:** Takafumi Yorozuya, Atsushi Saito, Tatsuya Sato, Tomoyuki Takahashi, Masami Kameda, Miki Yamaguchi, Yuji Sakuma, Nobutoshi Ichise, Noritsugu Tohse, Masato Furuhashi, Hirofumi Chiba

**Affiliations:** 1 Department of Respiratory Medicine and Allergology, Sapporo Medical University School of Medicine, Sapporo, Japan; 2 Division of Cellular Physiology and Signal Transduction, Department of Physiology, Sapporo Medical University School of Medicine, Sapporo, Japan; 3 Division of Cardiovascular-Kidney-Metabolic Medicine, Department of Internal Medicine, Sapporo Medical University School of Medicine, Sapporo, Japan; 4 Respiratory Medicine, JR Sapporo Hospital, Sapporo, Japan; 5 Department of Molecular Medicine, Institute for Immunology, Sapporo Medical University School of Medicine, Sapporo, Japan; 6 Department of Health and Nutrition, Faculty of Human Sciences, Hokkaido Bunkyo University, Eniwa, Japan

**Keywords:** mitochondria, alveolar macrophages, interstitial lung diseases, extracellular flux analyzer, translational study

## Abstract

**Background:**

Intracellular metabolism is essential for macrophage function. However, the association between the metabolic profile of alveolar macrophages (AMs), which are responsible for pulmonary innate immune responses, and the progression of pulmonary fibrosis in humans remains unclear.

**Methods:**

This exploratory study investigated whether mitochondrial bioenergetics in bronchoalveolar lavage (BAL)-derived AMs can distinguish interstitial lung disease (ILD) from non-ILD conditions. A total of 12 patients undergoing diagnostic BAL were analyzed (ILD, *n* = 7; non-ILD, *n* = 5). CD11c^+^ AMs were isolated and analyzed using an extracellular flux analyzer to quantify metabolic parameters. Principal component analysis (PCA) and penalized logistic regression were used for multivariate discrimination.

**Results:**

The AMs in ILD showed reduced ATP-linked respiration (3.55 vs. 10.67 pmol/min/10^5^ cells, *p* = 0.003) and coupling efficiency (10.55% vs. 25.81%, *p* = 0.018) compared with non-ILD AMs. PCA using all four metrics distinguished ILD from non-ILD AMs. Logistic regression classified subjects with 83% cross-validated accuracy. ATP-linked respiration and coupling efficiency were independent predictors under Firth correction. Collectively, AMs from ILD cases exhibited mitochondrial dysfunction, particularly reduced ATP-linked respiration.

**Conclusion:**

This bioenergetic profile may offer preliminary insights into mitochondrial bioenergetic alterations in AMs from ILDs and their potential relevance to disease characterization. Larger longitudinal studies are warranted to validate these findings and their clinical utility.

## Introduction

Interstitial lung diseases (ILDs) are a diverse group of disorders characterized by aberrant wound healing that leads to progressive pulmonary fibrosis (1). Although antifibrotic therapies can slow functional deterioration, the median survival for idiopathic pulmonary fibrosis (IPF), the prototypic ILD, remains poor at only 3–5 years (2, 3), highlighting the need for mechanistic biomarkers and novel therapeutic strategies. Dysregulated innate immunity and extracellular matrix (ECM) remodeling have been implicated as convergent drivers of fibrosis (4, 5), but the metabolic basis of these processes in humans remains incompletely understood.

Alveolar macrophages (AMs) are the predominant resident immune cells in the lung that serve as the first line of defense against inhaled pathogens and particulates (6). Among the various methods for evaluating macrophage function, metabolic profiling using an extracellular flux analyzer has demonstrated relevance in macrophage activation and inflammation (7, 8). Alterations in the metabolic phenotypes of AMs have been reported to contribute to the progression of certain lung diseases (9, 10). Moreover, mitochondrial dysfunction, a central regulator of cellular metabolism, has been implicated in the pathogenesis of ILD across multiple lung cell types (4, 11). While mitochondrial dysfunction has been reported in multiple lung cell types in ILD, the mitochondrial bioenergetic profile of human BAL-derived AMs remains less well characterized.

To address this gap, this exploratory study characterized the metabolic profile of bronchoalveolar lavage (BAL)-derived AMs from patients with ILDs, focusing on mitochondrial respiratory function using an extracellular flux analyzer. Mitochondrial oxygen consumption parameters were compared between patients with ILDs and those with other respiratory diseases, and their ability to discriminate ILD status was evaluated. This study aims to provide foundational insights for future translational research.

## Materials and methods

### Ethical approval

This study was conducted in accordance with the Declaration of Helsinki and was approved by the ethics board of Sapporo Medical University Hospital (#342-200). All participants were informed about the study protocols and objectives, and written informed consent was obtained.

### Patient enrollment and clinical data collection

This study prospectively enrolled patients with ILDs (ILD group) and other respiratory diseases (non-ILD group) who underwent BAL as part of their standard medical diagnosis or treatment at Sapporo Medical University Hospital and JR Sapporo Hospital between May and October 2023. The ILD group consisted of IPF (*n* = 1), pleuroparenchymal fibroelastosis (PPFE, *n* = 1), connective tissue disease-associated interstitial lung disease (CTD-ILD, *n* = 3), and unclassifiable ILD (*n* = 2). The non-ILD group included five cases subjected to BAL for other indications without evidence of pulmonary fibrosis on high-resolution computed tomography (HRCT). Disease severity was assessed using the Gender, Age, and Physiology (GAP) score (12). Physical information, pulmonary function tests [i.e., percent predicted forced vital capacity (%FVC) and diffusing capacity of the lung for carbon monoxide (%DLCO)], and blood tests [i.e., surfactant protein-D (SP-D) and Krebs von den Lungen-6 (KL-6)] were obtained from electronic medical records at hospitalization or the nearest date to BAL.

### BAL procedure

BAL was performed by respiratory medicine specialists. A total of 150 mL of saline was administered into the bronchi in three aliquots of 50 mL each, with each instillation followed by the maximum possible aspiration. Specimens from which at least 50 mL of fluid was recovered were included in the analysis according to the protocol. BAL recovery rate and BAL cell concentration showed overlapping distributions between ILD and non-ILD groups (median recovery rate 0.63 vs. 0.57; median cell concentration 2.42 vs. 0.67 × 10^5^/mL). Metabolic analyses were performed using subsequently purified CD11c^+^ macrophages.

### Macrophage separation

The BAL fluid samples were kept on ice and processed within 2 hours. The cells were centrifuged, treated with a hemolytic agent, and washed with phosphate-buffered saline (PBS). AMs were labeled with CD11c using a Biotin-Antibody Cocktail (#337232, BioLegend, USA), followed by incubation with Anti-Biotin MicroBeads (#130-090-485, Miltenyi Biotec, Germany). Magnetic separation was performed using a magnetic-activated cell sorting (MACS) column (#130-042-201, Miltenyi Biotec), where unlabeled cells were removed through sequential washes, and labeled AMs were eluted. A NovoCyte Flow Cytometer (Agilent Technologies, USA) was used to confirm AM purity with an anti-human CD11c antibody (clone Bu15, #337213, BioLegend) and a mouse IgG1 isotype control (clone MOPC-21, #981802, BioLegend). A representative result of the flow cytometry is shown in Supplementary Figure S1. Only AMs with a purity of at least 90% were used in subsequent experiments.

### Extracellular flux assay

The oxygen consumption rate (OCR) of isolated AMs was measured using a Seahorse XFe96 Bioanalyzer (Agilent Technologies) according to the manufacturer’s instructions. A total of 1.0 × 10^5^ AMs in 50 μL PBS were seeded onto a pre-warmed 96-well Seahorse XF poly-D-lysine (PDL)-coated microplate (#103798-100, Agilent Technologies), which was then incubated in a CO₂-free incubator at 37 °C for 45 min to allow cell attachment. Afterward, the PBS was carefully removed, and 180 μL of pre-warmed Seahorse XF DMEM assay medium containing 5.5 mM glucose, 1.0 mM sodium pyruvate, 2.0 mM glutamine, and 5.0 mM HEPES (pH 7.4) was added. The OCR and extracellular acidification rate (ECAR) were simultaneously measured at baseline after sequential injections with oligomycin (1.0 μM; #75351, Sigma-Aldrich, USA), carbonyl cyanide p-trifluoromethoxyphenylhydrazone (FCCP, 2.0 μM; #C2920, Sigma-Aldrich), and a mixture of rotenone (1.0 μM; #R8875, Sigma-Aldrich) and antimycin A (1.0 μM; A8674, Sigma-Aldrich). To minimize inter-assay variability, background correction and standardized assay conditions were applied across Seahorse experiments. The OCR and ECAR data were normalized solely to cell number in the present study. Protein-based normalization was not performed because PDL-coated plates were used for AM attachment, which may interfere with post-assay protein quantification, especially in BAL samples with low cell numbers.

### Calculation of metabolic indices

The metabolic indices of the OCR using the extracellular flux analyzer were calculated as previously reported with modifications (13). Basal respiration: OCR_(Baseline)_ – OCR_(R/A)_, ATP-linked respiration: OCR_(Baseline)_ – OCR_(Oligo)_, Proton leak: OCR_(Oligo)_ – OCR_(R/A)_, Coupling efficiency: ATP-linked respiration divided by basal respiration multiplied by 100, Maximal respiration: OCR_(FCCP)_ – OCR_(R/A)_, Spare respiratory capacity: OCR_(FCCP)_ – OCR_(Baseline)_, Non-mitochondrial respiration: OCR_(R/A)_, Basal ECAR: ECAR_(Baseline)_.

### Statistical analysis

Data are expressed as mean ± standard deviation (SD) for normally distributed variables and median [interquartile range] for non-normally distributed variables. Group differences were assessed using unpaired t-tests or Mann–Whitney U tests as appropriate. Principal component analysis (PCA) was used for dimensionality reduction and visualization. Prior to PCA, all input variables were standardized using *Z*-score normalization to account for differences in scale and ensure appropriate weighting. L2-regularized logistic regression and Firth’s penalized logistic regression were applied to evaluate the classification performance and variable contributions. A *p*-value of <0.05 was considered significant. Analyses were performed using GraphPad Prism 9 software (GraphPad, Inc., San Diego, CA, USA), Python (v3.11) and R (v4.3.2).

## Results

### Patient characteristics

Patient characteristics are shown in Table 1. The ILD group had a higher mean age compared to the non-ILD group (67.4 ± 12.4 vs. 55.2 ± 10.0 years). The ILD group had a lower proportion of females, whereas the non-ILD group was exclusively female (3/7 [42.9%] vs. 5/5 [100%]). Smoking history, assessed in pack-years, was also higher in the ILD group than in the non-ILD group (23.0 ± 17.4 vs. 7.9 ± 14.1 pack-years). Pulmonary function tests revealed that %FVC (95.7 ± 30.3 vs. 107.9 ± 12.0) and %DLCO (66.9 ± 23.2 vs. 83.1 ± 13.4) were both lower in the ILD group compared with the non-ILD group, while SP-D (315.3 ± 220.4 vs. 91.1 ± 47.4 ng/mL) and KL-6 (1501.0 ± 1855.5 vs. 431.0 ± 229.0 U/mL) were both higher in the ILD group.

**Table 1:** Baseline characteristics of the recruited subjects.

Variables	Total (*n* = 12)	ILDs (*n* = 7)	Non-ILDs (*n* = 5)
Age (years)			
Mean	62.3 ± 12.7	67.4 ± 12.4	55.2 ± 10.0
Range	42–82	45–82	42–69
Gender			
Male	4	4	0
Female	8	3	5
Smoking (pack-years)	16.7 ± 17.3	23.0 ± 17.4	7.9 ± 14.1
Pulmonary function test			
%FVC	100.1 ± 25.1	95.7 ± 30.3	107.9 ± 12.0
%DLCO	72.8 ± 21.0	66.9 ± 23.2	83.1 ± 13.4
Biochemical data			
SP-D (ng/mL)	233.8 ± 206.4	315.3 ± 220.4	91.1 ± 47.4
KL-6 (U/mL)	1111.9 ± 1540.4	1501.0 ± 1855.5	431.0 ± 229.0

Variables are expressed as number (%), means ± SD. ILD, interstitial lung disease; %FVC, forced vital capacity percent predicted; %DLCO, diffusing capacity of carbon monoxide percent predicted; SP-D, surfactant protein-D; KL-6, Krebs von den Lungen-6.

### Mitochondrial respiratory function of AMs

**Figure 1:**
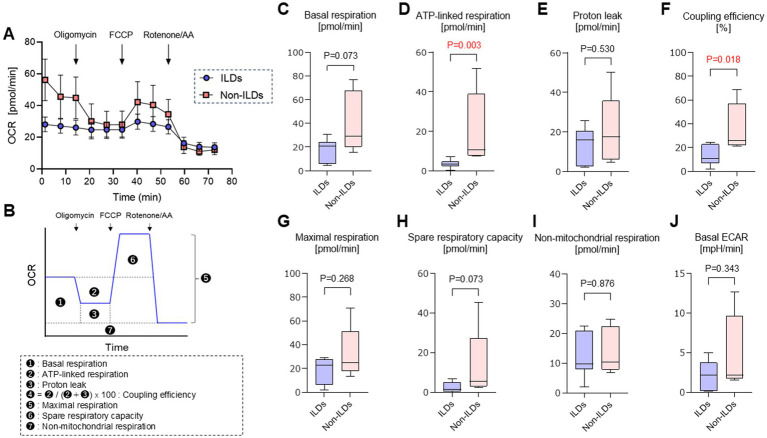
Mitochondrial respiration in AMs assessed by extracellular flux analysis. **(A)** Representative trace of oxygen consumption measurement in AMs obtained from patients with ILDs and those with non-ILDs. **(B)** Each mitochondrial function parameter was schematically calculated based on OCR measurements. **(C)** Basal respiration. **(D)** ATP-linked respiration. **(E)** Proton leak. **(F)** Coupling efficiency. **(G)** Maximal respiration. **(H)** Spare respiratory capacity. **(I)** Non-mitochondrial respiration. **(J)** Basal ECAR. ILD, interstitial lung disease; OCR, oxygen consumption rate; ECAR, extracellular acidification rate; FCCP, carbonyl cyanide *p*-trifluoromethoxyphenylhydrazone; AA, Antimycin A. Data are expressed as mean ± standard deviation for panel **(A)** and median [interquartile range] for panels **(C–J)**. *p* < 0.05 indicates statistical significance.

The results of the extracellular flux analysis of CD11c^+^ AMs isolated from BAL fluids and their metabolic parameters are shown in Figure 1. ATP-linked respiration was significantly lower in the ILD group (median: 3.55 vs. 10.67 pmol/min/10^5^ cells, *p* = 0.003). Moreover, coupling efficiency, which represents the proportion of respiration used for ATP synthesis, was also significantly reduced in the ILD group (10.5% vs. 25.8%, *p* = 0.018). Basal respiration and spare respiratory capacity were comparable between the ILD and non-ILD groups. Similarly, proton leak, maximal respiration, and non-mitochondrial respiration showed no significant differences between two groups. There were also no statistical differences in glycolytic indices between the two groups.

### Multivariate pattern recognition

**Figure 2:**
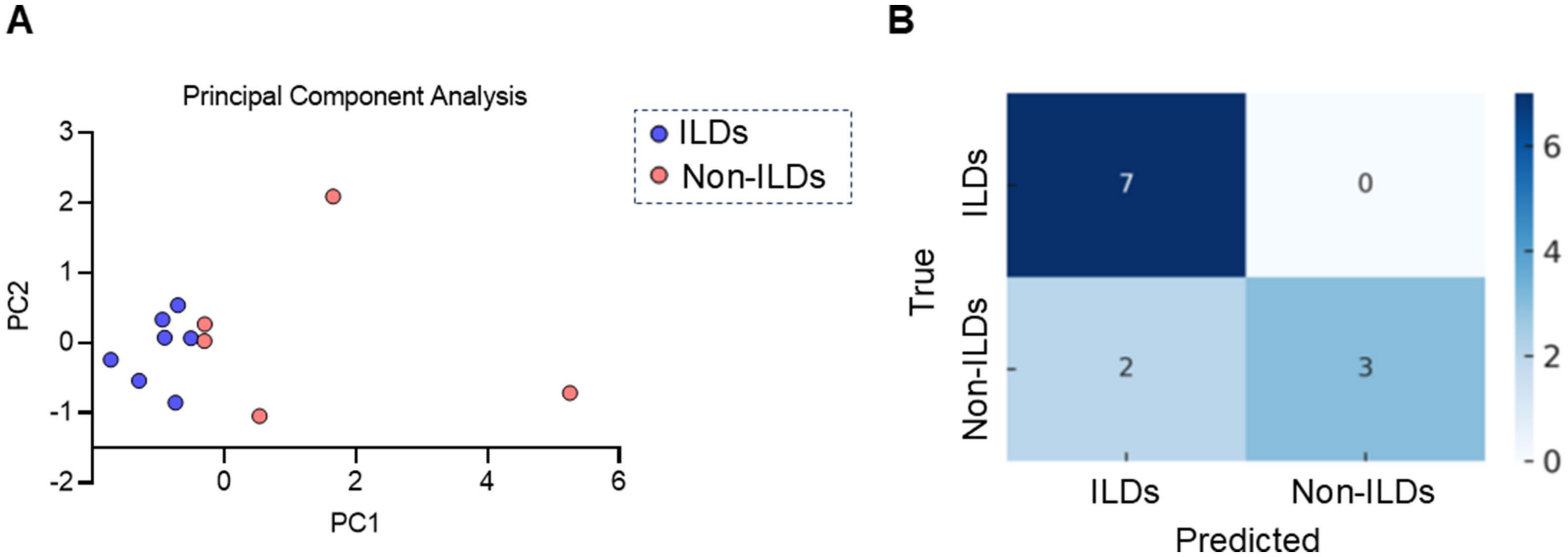
Multivariate classification analysis based on mitochondrial functions in AMs. **(A)** A map of PCA using four standardized mitochondrial parameters: ATP-linked respiration, coupling efficiency, basal respiration, and spare respiratory capacity. **(B)** Confusion matrix of the L2-regularized logistic regression evaluated via leave-one-out cross-validation. True labels are shown on the vertical axis, while predicted labels are shown on the horizontal axis. PCA, principal component analysis; ILD, interstitial lung disease.

To better understand how mitochondrial metabolic features differentiate ILD from non-ILD conditions, PCA was performed using four key parameters: ATP-linked respiration, coupling efficiency, basal respiration, and spare respiratory capacity (Figure 2). The first two principal components accounted for 72% of the total variance, with PC1 (48%) mainly driven by coupling efficiency and ATP-linked respiration, and PC2 (24%) mainly by basal respiration and spare respiratory capacity. In the PC1-PC2 plane, the ILD and non-ILD cases formed largely distinct clusters (Figure 2A). To further evaluate the classification performance, L2-regularized logistic regression using the same four variables showed an accuracy of 83% under leave-one-out cross-validation (Figure 2B).

To address potential small-sample bias and separation issues, we also employed Firth’s penalized logistic regression. As shown in Table 2, coupling efficiency (*β* = −1.98; 95% CI: −5.20 to −0.44, *p* = 0.012) and ATP-linked respiration (β = −1.25; 95% CI: −3.90 to −0.12, *p* = 0.031) remained significant independent predictors of ILD. In contrast, basal respiration and spare respiratory capacity were not statistically significant. These findings suggest that specific forms of mitochondrial dysfunction in AMs can distinguish ILD from other respiratory diseases.

**Table 2:** Coefficient estimates from Firth’s logistic regression model for ILD classification.

Variable	Regression coefficients (*β*)	CI (lower)	CI (Upper)	*p* value
Intercept	−0.85	−3.2	1.1	0.39
ATP-linked respiration	−1.25	−3.9	−0.12	0.03
Coupling efficiency	−1.98	−5.2	−0.44	0.01
Basal respiration	−0.78	−2.6	0.45	0.21
Spare respiratory capacity	−0.34	−1.9	0.92	0.38

ILD, interstitial lung disease; CI, 95% confidence interval.

### Correlation between disease severity and mitochondrial respiratory function of AMs

Finally, to explore the relationship between disease severity and metabolic profile, patients were divided into two groups based on GAP scores: 0–2 (lower risk, *n* = 4) and 3–4 (higher risk, *n* = 3). Subgroup analysis showed no significant differences in mitochondrial parameters, including basal, ATP-linked, maximal respiration, and spare respiratory capacity, between the groups (Supplementary Figure S2). These findings suggest that the mitochondrial bioenergetic profile of AMs is not clearly associated with disease severity as defined by the GAP score, although the limited sample size warrants caution in interpreting the results.

## Discussion

AMs serve as the first line of defense in the lung, organizing essential functions such as phagocytosis, surfactant recycling, and cytokine release, which are energy-dependent processes driven by mitochondrial function and redox signaling (14, 15). Recent experimental studies have shown that altered AM metabolism, including shifts in oxidative phosphorylation and glycolysis, can modulate inflammatory responses and lung fibrosis (16–18). Consistently, this study demonstrated that ATP-linked respiration and coupling efficiency were significantly lower in AMs isolated from patients with ILDs compared to those with non-ILDs, despite the heterogeneity of the ILD group, which included IPF, PPFE, CTD-ILD, and unclassifiable ILD. Taken together, mitochondrial respiratory dysfunction in BAL-derived AMs from patients with ILD may underlie the functional impairment of AMs observed in ILD.

Impaired oxidative phosphorylation and reduced mitochondrial biogenesis in various constituent cells of the lung have been previously reported in ILD (4, 11, 19, 20). Reduced ATP production in AMs may compromise energy-dependent processes such as efferocytosis, potentially contributing to secondary necrosis and sustained epithelial injury as suggested by prior studies (21). Energy-deprived AMs have been associated with increased expression of profibrotic mediators, including C-C Motif Chemokine Ligand 18 (CCL18), galectin-3, and transforming growth factor-*β* (TGF-β) activators, which are linked to disease progression and poor prognosis in IPF (22, 23). Furthermore, studies in murine models of lung fibrosis have shown that the pharmacological restoration of mitochondrial biogenesis in AMs attenuates collagen accumulation and improves lung compliance (19). Taken together, previous evidence and our present findings suggest an association between AM bioenergetics and the development of lung fibrosis, although further mechanistic studies are needed to establish causality.

Our data also revealed a significant reduction in coupling efficiency in AMs from patients with ILDs, indicating a weakened link between electron flux and ATP synthesis. Such uncoupling has been associated with electron leakage and increased mitochondrial reactive oxygen species (mtROS) production in other experimental settings (24). Excess mtROS amplifies TGF-*β* signaling and promotes ECM deposition, while persistent oxidative stress can further damage the mitochondrial respiratory chain, creating a vicious cycle of mitochondrial dysfunction (11, 21). Although mtROS was not directly measured, the observed bioenergetic profile of AMs from ILD cases is consistent with the possibility that mitochondrial dysfunction is associated with oxidative stress-related processes in lung fibrosis. These observations align with the concept that AM mitochondria could function as a key redox-metabolic hub in fibrotic lung disease, although direct mechanistic validation is required. Notably, non-mitochondrial respiration was comparable between ILD and non-ILD AMs, suggesting that the contribution of non-mitochondrial sources of ROS, such as xanthine oxidoreductase or NADPH oxidases (25), is similar between the two groups. Thus, mitochondrial dysfunction in AMs may be associated with mtROS production, which can directly injure alveolar epithelial cells and activate canonical profibrotic pathways. Moreover, the metabolic reprogramming of AMs may skew their cytokine profile toward profibrotic mediators, thereby promoting fibroblast proliferation and ECM accumulation (23, 26).

In the present study, AMs from ILD cases showed no increase in basal ECAR, indicating the absence of a metabolic shift toward glycolysis. Consequently, these cells did not exhibit classical M1 or M2 polarization phenotypes, which are associated with glycolytic and oxidative profiles, respectively (27). This atypical metabolic phenotype may reflect macrophage heterogeneity beyond the conventional M1/M2 framework. Recent studies have demonstrated that tissue macrophages, including alveolar macrophages, display diverse activation states and metabolic programs influenced by their origin, microenvironment, and oxygen availability (28–31). Such cellular diversity, including contributions from recruited monocyte-derived macrophages, may have influenced the mitochondrial bioenergetic profiles observed in the present study. Alternatively, the lack of basal ECAR elevation despite impaired mitochondrial respiration may indicate a failure to activate compensatory glycolysis or a shift toward other pathways such as fatty acid oxidation, which regulates macrophage function in various conditions (32). Further studies assessing substrate utilization in AMs may help clarify the comprehensive metabolic phenotypes in ILDs.

By integrating the four mitochondrial functional parameters into a penalized logistic regression model, we were able to distinguish ILDs from non-ILDs with high cross-validated accuracy. Thus, a composite metabolic signature in BAL-derived AMs could serve as a promising biomarker for ILDs. From a therapeutic perspective, strategies aimed at restoring mitochondrial coupling or biogenesis in AMs are promising approaches for intervention, but their clinical efficacy remains to be established (19, 26). Differences in metabolic parameters across GAP scores were not evident, although the small number of cases warrants caution in interpreting this negative finding.

This study has several limitations. First, the small sample size reflects exploratory and pilot design of this study. In addition, the ILD group comprised heterogeneous disease entities with distinct etiologies, and meaningful subtype-specific or classification analyses were not feasible due to the limited sample size. Furthermore, the limited sample size precluded evaluation of differences in AM phenotypes by age (33, 34), sex (35, 36), smoking history (37), and comorbidities (38), all of which are known to influence immune cell metabolism and mitochondrial function. Accordingly, the results of statistical comparisons, PCA, and logistic regression should be interpreted with caution, as model stability, generalizability, and susceptibility to overfitting cannot be reliably assessed in such a small pilot study. Nevertheless, a strength of this study lies in the use of freshly obtained patient specimens and highly purified AMs. Second, as obtaining BAL samples from healthy individuals is ethically challenging in Japan, all samples were originally obtained for clinical purposes, and subsequently utilized for research with informed consent. The non-ILD group included individuals with other pulmonary diseases, serving as a clinical comparison group, which may have affected AM mitochondrial function. Third, due to limited cell yields from BAL, AM viability could not be assessed prior to Seahorse assays, representing an important limitation of this study. However, preserved reductions in OCR following rotenone and antimycin A suggest intact electron transport chain activity. Fourth, in addition to the limitations of CD11c-based cell separation, the potential functional effects of magnetic bead-based isolation, including macrophage activation (39), should be considered, as these factors may have influenced the results. Finally, molecular mechanisms underlying AM mitochondrial dysfunction were not addressed. Future research should explore factors such as mitochondrial DNA mutations, altered dynamics, and quality control.

## Conclusion

Mitochondrial dysfunction in AMs, characterized by reductions in ATP-linked respiration and coupling efficiency, is associated with ILDs. Metabolic profiling of BAL-derived AMs was able to distinguish between ILD and non-ILD cases, suggesting its potential relevance to disease characterization and future therapeutic targeting.

## Data Availability

The original contributions presented in the study are included in the article/Supplementary material, further inquiries can be directed to the corresponding author.
