# Supplementary figures and images for: Mitochondrial respiratory dysfunctions of alveolar macrophages in interstitial lung disease: an exploratory study of bioenergetic and clinical links
Source: Front Med (Lausanne). 2026 Jan 22;12:1719710. doi: 10.3389/fmed.2025.1719710 (PMC12872845; doi:10.3389/fmed.2025.1719710)

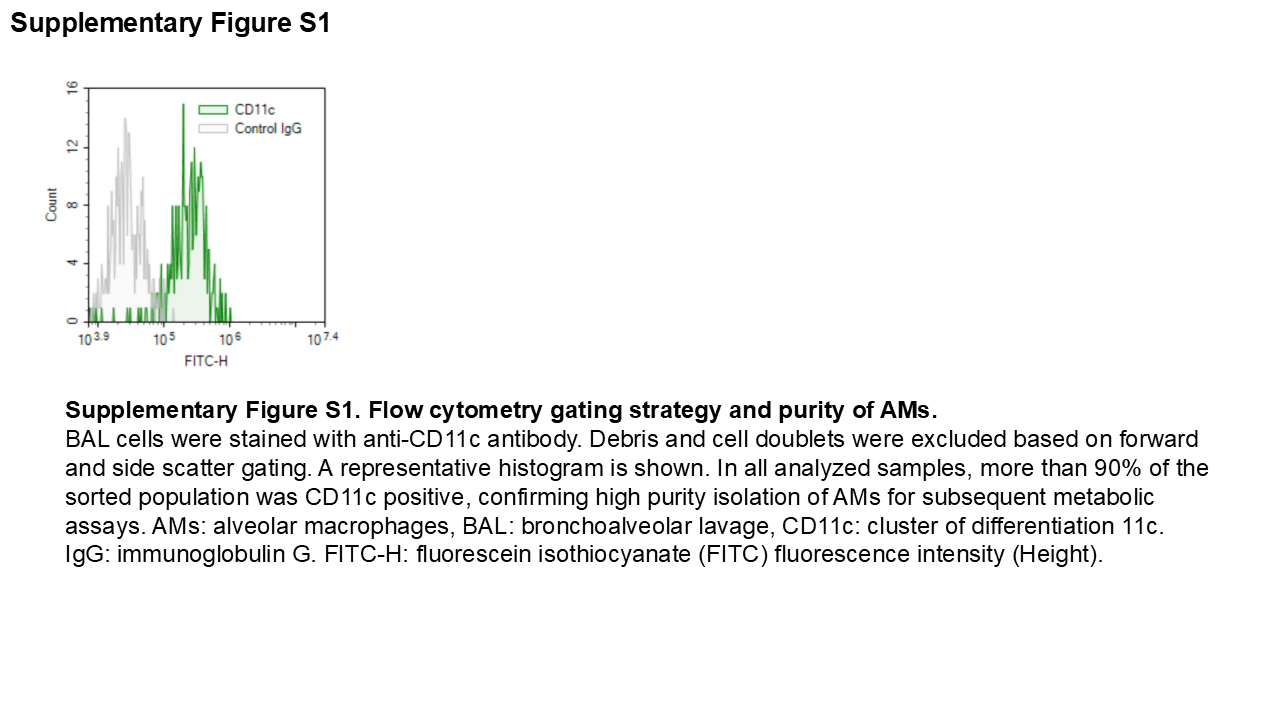

Supplement: Supplementary file 1 [file Image_1.tif]

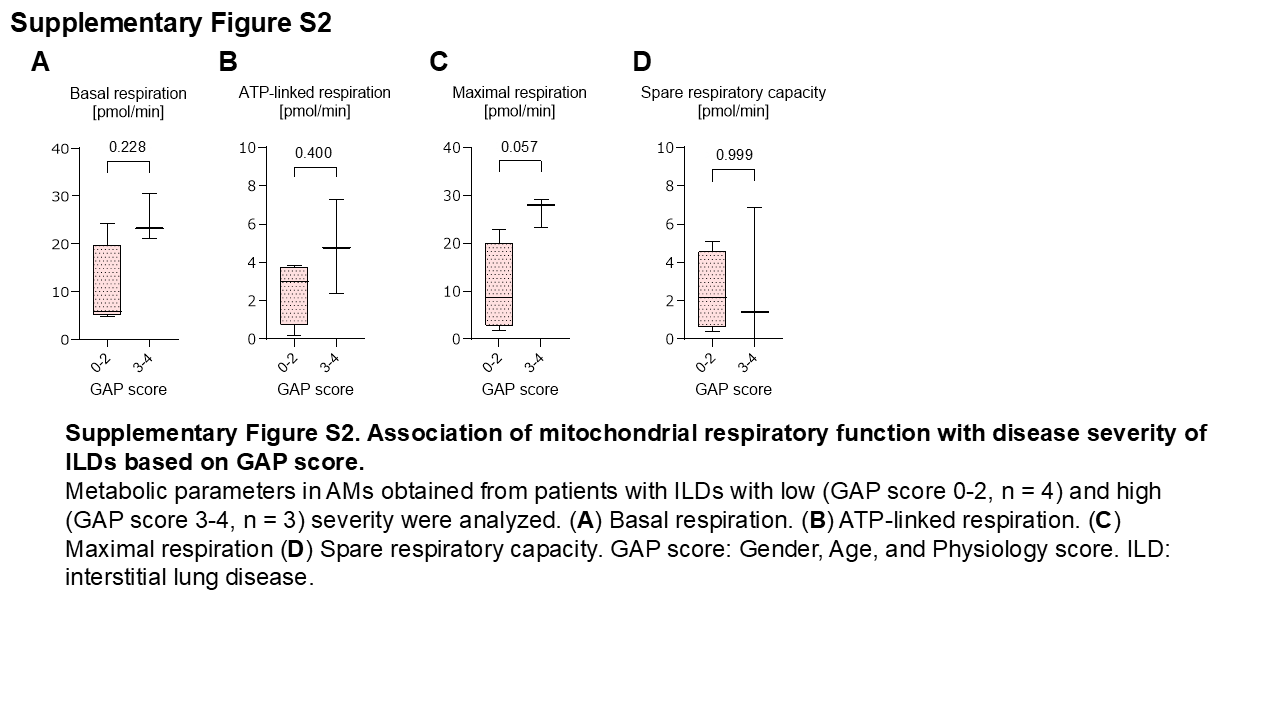

Supplement: Supplementary file 2 [file Image_2.tif]
